# The Role of Interventional Radiology in Treating Complications following Liver Transplantation

**DOI:** 10.1155/2013/696794

**Published:** 2012-12-03

**Authors:** Homoyoon Mehrzad, Kamarjit Mangat

**Affiliations:** Interventional Radiology Department, Queen Elizabeth Hospital, University Hospitals Birmingham, Birmingham B15 2WB, UK

## Abstract

Liver transplantation (LT) is used to treat both adult and pediatric patients with end-stage liver disease or acute liver failure. It has become more prevalent as both the surgical technique and postoperative care have improved resulting in a reduced morbidity and mortality. As a result, there are more patients surviving longer after liver transplantation. Despite this, there remain serious complications from the procedure that have a significant outcome on the patient and may result in retransplantation. At the same time, there have been significant advances in the field of interventional radiology both in terms of technology and how these apply to the patients. In this paper, we review the commonest complications, diagnostic tests, and interventional management options available.

## 1. Introduction

Liver transplantation (LT) is used to treat both adult and pediatric patients with end-stage liver disease or acute liver failure. It has become more prevalent as both the surgical technique and postoperative care have improved resulting in a reduced morbidity and mortality. As a result, there are more patients surviving longer after liver transplantation. Despite this, there remain serious complications from the procedure that have a significant outcome on the patient and may result in retransplantation. At the same time, there have been significant advances in the field of interventional radiology both in terms of technology and how these apply to these patients. The main advantage is the ability to treat the common complications via a percutaneous minimally invasive manner reducing the need for further surgery with the aim of preserving the function of the transplanted liver. As a result, interventional radiologists have become an important member in the multidisciplinary transplantation team. The commonest method of liver transplantation is an orthotopic type (OLT) where the donor organ is placed in the same anatomical location as the original. The techniques described in this paper apply to deceased donor LT and living related LT (LRLT) both in the adult and pediatric population. In this paper, we aim to review the common complications following liver transplantation, the diagnostic tools available, and the available interventional treatments including potential complications. This paper is a review of the range of procedures offered by the interventional radiologist and is a mix of our experience in one of the largest transplant centers in Europe—at our institutes, we currently perform over 180 adult and pediatric liver transplants per year—and a review of the related literature. The paper is aimed as a reminder for all those clinicians who may be dealing with posttransplant patients and highlights the alternative options available as compared to surgery.

## 2. Methods: Diagnostic Tools Available for Investigation of Liver Transplant Patients

**Figure 1:**
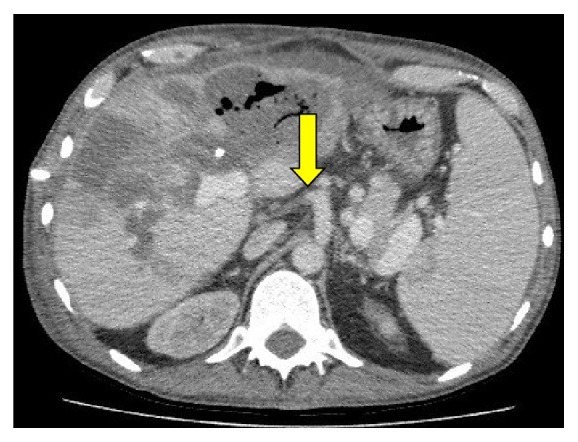
CT scan in a patient following liver transplantation who developed liver abscess. The CT scan demonstrated an occluded transplanted hepatic artery (HAT) arising from the celiac artery (yellow arrow).

Several of the common complications can present in a similar manner with reduced liver function and deranged liver function tests. It is therefore important that a timely and accurate diagnosis is made in order to initiate the correct treatment. Ultrasound (US) with Doppler studies remains largely the first line tool in investigating the LT both in terms of assessing the vascular and biliary systems. It is also routinely used for followup and screening in these patients. Computer tomography (CT) and CT angiography (CTA) are helpful for further assessment of the liver, the anatomical structures, and vascular structures (Figure 1). Magnetic resonance imaging (MRI) and magnetic resonance cholangiopancreatography (MRCP) are used as problem solving tools in the assessment of the liver parenchyma and biliary systems, respectively. 

Despite these available tools, there are situations when the altered liver function cannot be explained by these tests, and a diagnostic liver biopsy is required to be performed. The two techniques are either via an ultrasound- (US-) guided percutaneous or a transjugular liver biopsy (TJLBx). The latter is reserved for patients with severe coagulopathy or a large amount of ascites. 

## 3. Treatment Options Available

### 3.1. Vascular Complications

Vascular complications following liver transplantation remain a significant problem resulting in increased mortality and morbidity in this group of patients. The majority of vascular problems arise within the first 3 months following transplantation [1]. The commonest complication is with the hepatic artery, but problems also arise within the hepatic vein, portal vein, and inferior vena cava (IVC). The clinical presentation of vascular complication can be indistinguishable from other liver transplant related complications.

#### 3.1.1. Hepatic Artery Stenosis/Thrombosis (HAS/HAT)

Hepatic artery complications have been reported in 4%–25% of patients [2, 3] with life-threatening hepatic artery thrombosis (HAT) noted in 3%–9% of patients [1, 4, 5]. The commonest surgical anastomosis performed is an end to end between the hepatic arteries, but if this is not technically possible, there are several alternatives, and therefore, knowledge of the surgical technique involved is crucial prior to investigation [2].

Risk factors for HAT include surgical technique, ischemic reperfusion injury, small donor artery, hepatic artery stenosis (HAS), and rejection [1].

**Figure 2:**
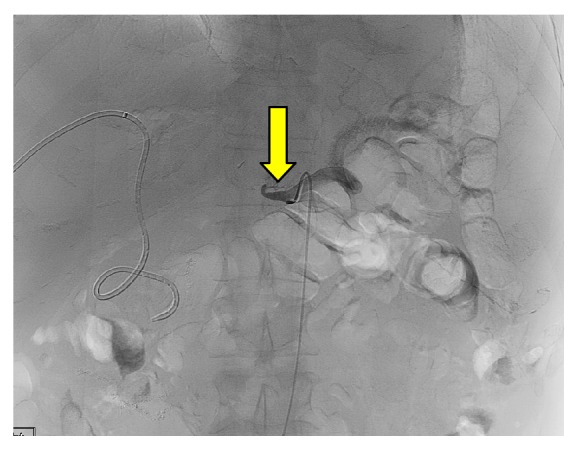
A celiac artery angiogram through a catheter demonstrating hepatic artery thrombosis (HAT) normal flow within the splenic artery is noted (yellow arrow). An internal/external drain within the transplanted liver was previously placed for biliary stenosis.

If HAT or HAS is suspected, this is initially assessed by noninvasive US Doppler or CTA, and if there is ongoing suspicion, then a selective catheter angiogram is performed to confirm the diagnosis and also treat any underlying cause (Figure 2). This is usually performed via a transfemoral arterial approach.

**Figure 3:**
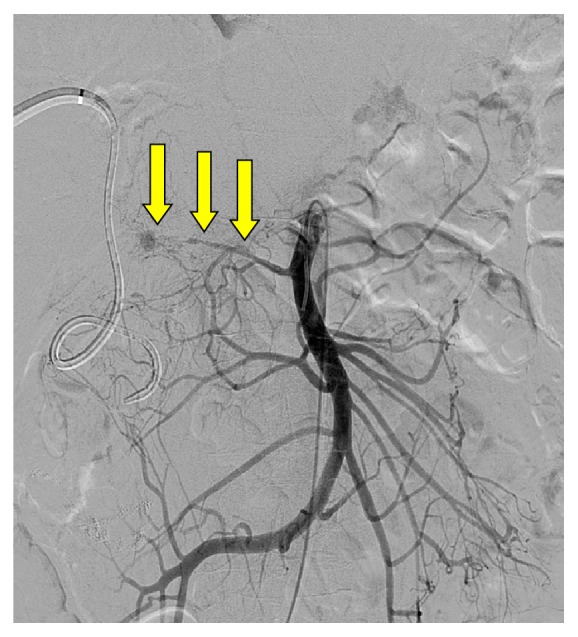
A superior mesenteric artery (SMA) angiogram through a catheter in a patient with the transplanted hepatic artery attached to the recipient's SMA. This demonstrates a hepatic artery stenosis (HAS). Due to the long-segment stenosis and small caliber, an angioplasty was not possible (yellow arrows).

In the case of HAS, the angiogram is performed and an angioplasty can be performed to treat the stenosis (Figure 3). The technique is similar to angioplasty in other parts of the vascular system. A small dose of intraarterial heparin and GTN is given to prevent further thrombosis and vascular spasm, respectively [2]. Stenting of the stenosis is reserved for resistant arterial stenosis where angioplasty alone is not successful. Angioplasty with or without stenting has now become a recognized treatment option in these patients with a high technical success rate reported resulting in an improvement of the liver function tests with a long-term patency in excess of 2.5 years [6]. The option of repeated angioplasty remains if there is a recurrence of the HAS. Potential complications include dissection or rupture of the vessel, but the incidences of these are low in experienced centers. 

In early HAT following confirmation at the time of angiography, there is a role for selective percutaneous thrombolytic therapy and treatment of any underlying stenosis with angioplasty/stent insertion [1]. There is also the ability to treat any steal syndrome that may be the cause for the HAT. This involves percutaneous embolisation of the splenic or gastroduodenal arteries arising from the celiac trunk in order to divert flow into the hepatic artery and perfuse the LT [1]. Surgical thrombectomy or reimplantation should be reserved for cases where the percutaneous methods have failed. There is currently no published randomized clinical trial evidence on the use of percutaneous thrombolytic therapy with only case reports published. 

#### 3.1.2. Portal Vein Stenosis

Portal vein stenosis usually presents with the signs of portal hypertension which includes ascites, varices, or splenomegaly [1, 2]. Confirmation of a portal vein stenosis and portal vein pressure gradient is usually via a transhepatic portogram with a transjugular approach as an alternative approach. The transhepatic approach affords a greater degree of control. Treatment options for the stenosis include balloon venoplasty with noncovered stents reserved for recurrent or residual stenosis. The use of stents in the pediatric patients should be used with caution as the stent does not enlarge as the patient grows [2]. Success rates of up to 74% have been reported in the literature [1, 7, 8] with no randomized controlled data. Complications of the transhepatic approach include bleeding along the track, and therefore, embolisation with coils and gelfoam is routinely used. 

#### 3.1.3. Hepatic Vein Stenosis

Hepatic venous stenosis presents with hepatic congestion leading to ascites and altered liver function tests. The stenosis usually occurs at the site of the anastomosis rather than intrahepatic venous stenosis [1]. It is important that a detailed knowledge of the surgical technique and vascular anatomy is known prior to investigation as the approach undertaken can vary. If a piggy-back anastomosis technique has been performed, then a transjugular approach allows better access to the hepatic veins, whereas if a caval interposition graft technique has been performed, either a jugular or femoral approach can be used [2]. An alternative approach to the hepatic veins is via a transhepatic approach, but this is usually reserved in cases where the other approaches are not successful. Venography of each of the hepatic vein, is performed to exclude any stenosis. It also allows pressure gradient measurements across any stenosis. A pressure gradient differentiation of greater than 3 mmHg across the stenosis is deemed significant [9]. Options for treating the stenosis again include balloon venoplasty with metallic stenting reserved for residual or recurrent stenosis [9–11]. The use of stents in the pediatric population has to be used with caution.

#### 3.1.4. Inferior Vena Cava Stenosis (IVCS)

This is usually due to a stenosis at the site of anastomosis or in the IVC superior to the site of anastomosis. The cause is usually iatrogenic or related to scar formation [1]. Clinical manifestations are ascites, renal failure, lower limb swelling, or altered liver function tests. The management is similar to the other vascular complications mentioned. The initial approach is usually via a transfemoral approach which allows an IVC venography to be performed. If an area of stenosis is identified, then pressure measurements can be performed. If this is significant, then balloon venoplasty is performed with a low threshold to stent the IVC in cases of residual or recurrent stenosis [1, 2]. Complications of rupture and stent occlusion have been reported. 

### 3.2. Biliary Complications

Biliary reconstruction following liver transplantation may be complicated with multiple sites of anastomosis. Those that have several sites of anastomosis or patients with a partial liver transplant have a greater risk of a complication [1]. Complications include biliary strictures, bile leaks, bile stones, and biliomas and occur in between 10%–40% of patients. The clinical presentation of the biliary complications is often nonspecific with the majority occurring within the first 3 months. The usual sites of anastomosis are a duct-to-duct anastomosis or a Roux-en-Y choledochojejunostomy [1]. Knowledge of this helps to determine which is the most appropriate initial method to be used to treat any potential complication. Those with a duct-to-duct anastomosis usually will be treated by endoscopic retrograde cholangiopancreatography (ERCP), whereas those with a Roux-en-Y anastomosis will be treated via a percutaneous transhepatic cholangiography (PTC) approach [1].

#### 3.2.1. Biliary Strictures

**Figure 4:**
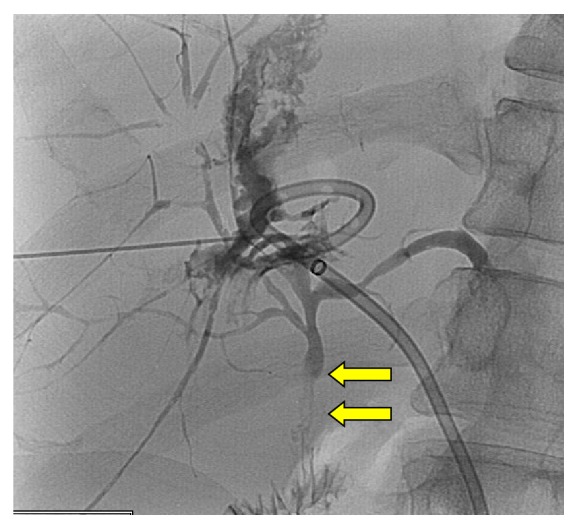
A PTC performed in a post-liver transplant patient with suspected biliary stenosis demonstrates a significant narrowing within the transplanted common bile duct (yellow arrows). A drainage catheter had previously been placed into a liver abscess.

This occurs at two sites either at the site of anastomosis, which is usually related to iatrogenic causes or scar formation, or within the intrahepatic biliary ducts, which is due to rejection, arterial insufficiency, or infections [1] (Figure 4). There is also the possibility of a combination of both. The role of PTC is to initially allow biliary drainage and subsequent biliary balloon dilatation to restore the normal drainage pathway. Following dilatation, a drainage catheter is placed across the stricture in order to prevent immediate restenosis. The process is usually repeated with increasing diameter balloon dilatation and increased size biliary drains placed across the stricture. This process results in a gradual dilatation of the biliary stricture. The long-term patency results have been reported at 73% at 1 year [12] and between 50%–60% at 5 years [13, 14]. Cutting balloons may be used in residual/recurrent stenosis. Metallic stents are reserved for cases where the stenosis is resistant to dilatation or those not fit for surgery [1]. Although PTC is usually reserved for cases with a Roux-en-Y anastomosis, it can be used in all cases where ERCPs are unsuccessful. The complications of PTC include haemobilia, intra/extrahepatic hematoma, and bacteremia. 

#### 3.2.2. Biliary Leaks

Biliary leaks are most common in the initial postoperative period with small leaks usually resolving without treatment. Larger leaks lead to increased morbidity and risk of sepsis. CT or ultrasound guided percutaneous drainage is the initial treatment option. PTC can be used to access the biliary system and place biliary drains to allow the biliary leak to resolve [1]. Combined PTC and endoscopic approach is reserved for difficult cases. 

## 4. Transjugular Intrahepatic Portosystemic Shunt (TIPS) in Liver Transplant Patients

**Figure 5:**
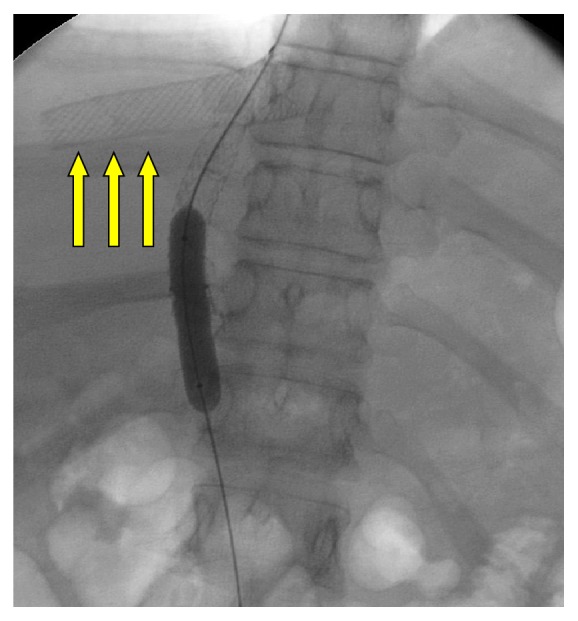
This image demonstrates a hepatic vein stent placed for hepatic venous stenosis in a liver transplant. The patient was undergoing a subsequent TIPS procedure.

**Figure 6:**
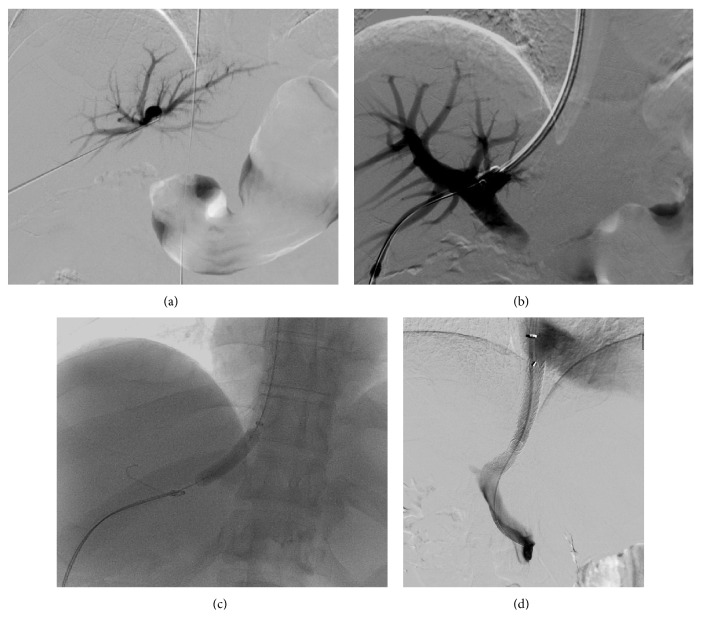
(a) A TIPS procedure in a liver transplant. A direct transhepatic puncture of the portal vein is initially performed. Contrast is injected through the needle to confirm the position. (b) A further puncture through the wall of the left portal vein into the hepatic vein is performed. A wire is passed up into the SVC, and a transjugular catheter is then advanced over the wire after it has been snared. (c) The remainder of the TIPS procedure is now performed via the routine transjugular approach. The track between the portal vein and hepatic vein is dilated with a balloon. (d) The TIPS stent is placed in situ, and contrast is injected into the portal vein to confirm flow through the stent and into the hepatic vein.

TIPS is increasingly used in the treatment of portal hypertension in patients with ascites or bleeding varices. There is, however, a limited experience of the role of TIPS in liver transplant patients with the main reason being the relatively rare occurrence of portal hypertension [15]. The underlying causes for the development of portal hypertension in these patients include recurrence of the underlying liver disease, organ size mismatch, increased vascular resistance or impaired venous outflow, and transplant rejection [16–18]. Therefore, the indications for TIPS are similar as those in nontransplant patients with the main significant difference being the altered anatomy. In patients that have had the piggy-back surgical anastomosis, the normal transjugular approach for TIPS may not be suitable. Therefore, a direct transhepatic puncture of the portal vein followed by puncture into the hepatic vein can be performed. A simultaneous transjugular approach is then performed, and the TIPS stent is then placed via the standard route (Figures 5 and 6). This approach provides a slightly different technical challenge than in nontransplant patients that require TIPS. Success rates of complete resolution or improvement of the ascites of between 50%–70% with 20% for variceal rebleeding have been quoted in the published data. Patency rates of greater than 80% have been reported at 1 year [19]. Complications include a procedural related mortality of approximately 2% and severe uncontrolled hepatic encephalopathy or hepatic insufficiency [19]. However, the interventional radiologist can either reduce or completely occlude the flow within the stent if required.

## 5. Miscellaneous Procedures

There are often other complications in post-liver transplant patients, these include hepatic artery pseudoaneurysm formation or arteriovenous malformations usually related to iatrogenic surgical or radiological procedure. These can be treated by percutaneous selective embolisation with embolic agents such as coils. 

## 6. Discussion

In this paper, we have reviewed both the diagnostic and percutaneous interventional radiological treatment options available in both adult and pediatric liver transplant patients. We have presented our experience in a busy transplant centre and reviewed the related literature. The main advantage to these procedures is that they can be performed via a percutaneous approach often negating the need for further surgery and extending the life of the liver transplant. Although these procedures do carry some morbidity- and mortality-related complications, they are generally less than related to surgery and have a proven track record. The role of the interventional radiologist in the management of these patients has increased and will continue to do so due to the developing technology and endovascular treatment options available. The increasing long-term survival of these patients will likely also lead to further interventional procedures due to recurrence of symptoms with repeated procedures on some patients. There is also an increasing experience in rarer procedures such as TIPS in post-liver transplant patients with these patients providing a differing challenge than the normal cohort as described in the paper. We hope that this paper will be a reminder for all those clinicians who may be dealing with posttransplant patients and highlight the alternative options available to these patients.
